# Significant relationships between a simple marker of redox balance and lifestyle behaviours; Relevance to the Framingham risk score

**DOI:** 10.1371/journal.pone.0187713

**Published:** 2017-11-06

**Authors:** Neda Seyedsadjadi, Jade Berg, Ayse A. Bilgin, Chin Tung, Ross Grant

**Affiliations:** 1 School of Medical Sciences, Faculty of Medicine, University of New South Wales, Sydney, New South Wales, Australia; 2 Australasian Research Institute, Sydney Adventist Hospital, Sydney, New South Wales, Australia; 3 Department of Statistics, Macquarie University, Sydney, New South Wales, Australia; 4 Sydney Adventist Hospital Clinical School, University of Sydney, Sydney, New South Wales, Australia; University of Nebraska-Lincoln, UNITED STATES

## Abstract

Oxidative stress has been closely linked to the progressive cell damage associated with emerging non-communicable disease (NCDs). Early detection of these biochemical abnormalities before irreversible cell damage occurs may therefore be useful in identifying disease risk at an individual level. In order to test this hypothesis, this study assessed the relationship between a simple measure of redox status and lifestyle risk factors for NCDs, and the population-based risk score of Framingham. In a cross-sectional study design, 100 apparently healthy middle-aged males (n = 48) and females (n = 52) were asked to complete a comprehensive lifestyle assessment questionnaire, followed by body fat percentage and blood pressure measurements, and blood collection. The ratio of plasma total antioxidant capacity to hydroperoxide (TAC/HPX) was used as an index of redox balance. One-way ANOVA and multiple linear regression analysis were performed to analyse the association between TAC/HPX, lifestyle components and other plasma biomarkers. The TAC/HPX ratio was higher in males compared to females (t_96_ = 2.34, P = 0.021). TAC/HPX was also lower in participants with poor sleep quality (t_93_ = 2.39, P = 0.019), with high sleep apnoea risk (t_62.2_ = 3.32, P = 0.002), with high caffeine (F(2, 93) = 3.97, P = 0.022) and red meat intake (F(2, 93) = 5.55, P = 0.005). These associations were independent of gender. Furthermore, the TAC/HPX ratio decreased with increasing body fat percentage (F(2, 95) = 4.74, P = 0.011) and depression score (t_94_ = 2.38, P = 0.019), though these associations were dependent on gender. Importantly, a negative association was observed between TAC/HPX levels and the Framingham risk score in both males (r(45) = -0.39, P = 0.008) and females (r(50) = -0.33, P = 0.019) that was independent of other Framingham risk score components. Findings from this study suggests that a relatively simple measure of redox balance such as the TAC/HPX ratio may be a sensitive indicator of redox stress, and may therefore serve as a useful biomarker for assessing an individual’s specific NCD risk linked to unhealthy lifestyle practices.

## Introduction

Non-communicable diseases (NCDs), such as cardiovascular disease, diabetes, cancer and degenerative dementias are among the leading causes of morbidity and mortality worldwide [1]. Although the importance of prevention has been highlighted in the literature for many years, integration of a successful preventive strategy in modern health care systems is still not apparent. Identification of groups at risk of NCDs is most often achieved through the use of population-based risk assessment tools [2, 3]. However, such tools do not specifically identify whether the individual’s biochemistry is actually experiencing damage. Unfortunately, for many, even being in the normal range of a particular health variable does not guarantee health, and there will still be a percentage of the population in this range who develop disease. The Framingham risk assessment tool is a widely used and validated population-based risk assessment tool for the prediction of coronary heart disease [2]. However, it has been shown to underestimate an individual’s risk and therefore is not able to identify actual progression to disease, only his ‘relative’ risk [4]. Conventional risk factors such as dyslipidaemia, hypertension, diabetes, and smoking cannot account for all cases of cardiovascular disease. The biochemical measures of total cholesterol (TC), low density lipoprotein cholesterol (LDL-C) and high density lipoprotein cholesterol (HDL-C) are now recognised as only being involved in downstream secondary pathologies, not as primary initiators of the disease process [5]. Thus, there are other less-conventional markers that are more indicative of the primary damage process and progression toward disease.

NCDs generally take many years to develop with clinical symptoms only becoming apparent after considerable damage has occurred in the target organ. Research now supports the argument that this damage is largely influenced by biochemical changes associated with redox imbalance producing a state of ‘oxidative stress’ (OS) [6, 7]. Chronic exposure to oxidative processes causes a subclinical state of progressive cell damage over time, which eventually results in selective systems failure and accompanying disease [8]. A simple biochemical marker of redox balance that reflects a person’s early biochemical shift toward disease during this preclinical stage would therefore be an advantage and may enable a more personalised and sensitive approach to preventive medicine besides conventional epidemiological classifications. Importantly, oxidative stress is recognised as a primary player in the early development of NCDs. In support of this concept, it has been shown that oxidized LDL is more atherogenic than native LDL [5]. Adding the lipid peroxidation biomarkers to the standard Framingham risk score has been shown to significantly improve its ability to predict coronary heart disease [9]. Therefore, including a measure of oxidative stress in any risk assessment tool may provide value that goes beyond the conventional risk factors for predicting disease.

As unhealthy lifestyles are widely accepted as primary driving forces behind the disease process for NCD’s [10], the major goal of public health efforts and current preventive health programs is modification of behaviours in those considered ‘at risk’ [11]. It is well known that lifestyle behaviours gradually shift the body’s biochemistry toward a diseased phenotype. If an unhealthy lifestyle persists, the resulting biochemical adaptation will, over a period of time, drive the body to a state of disease [12]. While often not picked up by routine pathology, such as cholesterol and glucose measurements, this chronic subclinical disease process may be detected via changes in the redox balance, an indicator of OS. If OS is a primary driver, the incidence of NCDs may arguably be reduced if it can be determined whether or not a state of redox imbalance or chronic OS exists as a consequence of an individual’s lifestyle behaviours. Therefore, the primary aims of this study were to firstly analyse the redox state of otherwise healthy subjects and assess its association with relevant lifestyle factors. Secondarily, we sought to determine whether the redox state also correlates with a population-based risk score (i.e. Framingham risk score). To the best of our knowledge, this is the first study to report an association between redox state and modifiable behaviours linked to a population-based risk assessment.

## Materials and methods

### Participants

This cross-sectional study included 48 males and 52 females aged between 40 and 75 years old and in general good health. Participants were excluded if they have any current microbial infection, have been diagnosed with cancer or been treated for cancer, have had major surgery in the past two years, have suffered from any form of neurodegenerative disease, inflammatory bowel disease, or a medically diagnosed liver or kidney disorder, have taken any diabetic, or thyroid medications, have taken any antibiotics, or anti-inflammatory medications, illicit drugs, supplements or any complementary medicines in the last two weeks prior to testing. After obtaining a written informed consent, participants were asked to complete a series of questionnaires for the assessment of their lifestyle behaviours. All the questionnaires (except dietary questionnaires) were completed online maximum two weeks before the blood collection and physiological assessments (hard copies of dietary questionnaires were completed on the same day as the blood collection and physiological assessments). Participants were asked to recall their lifestyle behaviours over the past four months. Blood collection, blood pressure measurement and body scanning for total body fat percentage analysis were all performed on the same day in a fasted state (about 12 hours). Participant recruitment was conducted at the University of New South Wales and Sydney Adventist Hospital campuses with ethical approval from the Adventist HealthCare Limited Human Research Ethics Committee, Sydney Adventist Hospital, Australia (HREC number: 2013–022).

### Questionnaires

The evaluation of sleep quality, sleep apnoea risk, depression score, physical activity level and sitting time, and caffeine intake was conducted by the validated questionnaires of Pittsburgh Sleep Quality Index (PSQI) [13], Berlin questionnaires [14], Depression Anxiety Stress Scale-21 (DASS-21) questionnaire [15], International Physical Activity Questionnaire-long version (IPAQ) [16], and Stanford questionnaire [17], respectively. For assessing alcohol intake and dietary intake, the validated 74-item Cancer Council Victoria Dietary Questionnaire for Epidemiological Studies Version 2 (DQES v2) [18] was used. In this study, average red meat intake was defined as the sum of the intake data for hamburger, beef, veal, lamb, pork, bacon, ham, salami and sausages. Furthermore, total carotenoid intake was calculated as the sum of the intake data for the most common dietary carotenoids including alpha-carotene, beta-carotene, beta-cryptoxanthin, lutein, zeaxanthin and lycopene.

### Biochemical analysis

Fasting plasma glucose (FPG), total cholesterol (TC), high density lipoprotein cholesterol (HDL-C) and triglyceride (TG) levels were measured by the enzymatic method on a Roche/Hitachi cobas c system (Sydney Adventist Hospital pathology laboratory). Low density lipoprotein cholesterol (LDL-C) levels were calculated by the Friedewald equation [19]. Plasma C—reactive protein (CRP) levels were quantified by Immunoturbidimetric assay on a Roche/Hitachi cobas c system (Sydney Adventist Hospital pathology laboratory). Plasma tumour necrosis factor- α (TNF-α), interleukin-1β (IL-1β) and IL-6 levels were measured using the MILLIPLEX^®^ MAP Human High Sensitivity T Cell Magnetic Bead Panel immunoassay (Merck KGaA, Darmstadt, Germany). Plasma reactive oxygen species (in the form of hydroperoxides), and total antioxidant capacity, were measured indirectly using the FORT (Free Oxygen Radical Test) and FORD (Free Oxygen Radicals Defence) colorimetric assays (CR3000, Callegari S.r.l., Catellani Group, Parma, Italy),as previously described [20].

### Calculation of redox balance

Redox balance was defined as the ratio of plasma total antioxidant capacity (TAC) levels (mmolTrolox equivalents/L) to plasma hydroperoxide (HPX) levels (mmol H_2_O_2_ equivalent/L) (TAC/HPX).

### Calculation of the Framingham risk score

The Framingham estimate of 10 year risk of coronary heart disease was derived from the Framingham risk score, based on age, systolic blood pressure, LDL-C and HDL-C concentrations and smoking by gender [2].

### Physiological analysis

Dual-energy X-ray absorptiometry (DXA) method was used to measure total body fat percentage by a Lunar iDXA (GE Healthcare, Madison, WI, USA) with an automatic total body scan mode. Participants were asked to change into a standard cloth gown and then were scanned by a trained operator as previously described [21].

Blood pressure was measured on both arms at rest (Omron SEM-2, Omron Healthcare Inc., Kyoto, Japan). Four measurements were conducted on each arm, without removal of blood pressure cuff, with 15 seconds interval between each measurement [22]. If the readings were different, the arm with the highest reading was used for the measurements thereafter and then, two lowest measurements for that arm were averaged [23].

### Statistical analysis

SPSS version 23 was used for statistical analysis. The Shapiro-Wilk and Kolmogorov-Smirnov tests were used to test the normality of the variables. After checking graphical displays and applying appropriate statistical rules, one outlier was removed for variables including alcohol intake, total energy intake, n-6 PUFA intake, n-3 PUFA intake, cholesterol intake, glycaemic index, retinol intake, vitamin E intake, IL-6 and TNF-α; two outliers were removed for variables including PSQI, depression score, caffeine intake, fruit intake, saturated fat intake, red meat intake, total carotenoid intake and IL-1β and three outliers were removed for variables including vitamin C intake, TG, CRP and Framingham risk score. Values were presented as means ± standard deviations. For the tertile categorization, cut-points were set for three equal groups in the related continuous variable and then the new categorical variables were defined based on the related cut-point values. Correlations between variables were determined using Pearson’s (*r* (n)) or Spearman’s (*r*_*s*_ (n)) correlation coefficients, as appropriate. The Student’s t-test was used to determine differences between groups with two categories.

One-way ANOVA followed by LSD post hoc test were used to determine significant differences between normally distributed groups with more than two categories. The Levene’s Test of Equality was applied to check homogeneity of variances between groups. If the variances of the groups were not homogenous, the Welch’s ANOVA followed by Games-howell post hoc test were performed. One-way ANCOVA was used for gender adjustment when analysing differences between categorical variables. Bonferroni adjustment was conducted to allow for the multiple comparisons involved.

Multiple linear regression analysis was performed to determine the association between the plasma TAC/HPX levels and FPG, total TC, LDL-C, IL-1β, and CRP after adjusting for age, gender and total body fat, and also to determine the association between the plasma TAC/HPX levels and Framingham risk score after adjusting for age, gender, LDL-C, HDL-C, and systolic and diastolic blood pressures. Adjusted *R*^*2*^ were reported. The Levene’s Test of Equality was used to check homogeneity of variances between groups. If the continues variables or the residuals of the regression were not normally distributed, log transformation or square roots of continues variables were investigated so that normality assumption of the residuals could be satisfied. P values less than 0.05 were considered statistically significant.

## Results

### Association between plasma TAC/HPX levels and various physiological and lifestyle components

Mean plasma TAC/HPX across categories of various physiological and lifestyle components are shown in Tables 1 and 2. Plasma TAC/HPX levels were significantly higher in males compared to females (t_96_ = 2.34, *P* = 0.021). There was no significant difference between age tertiles in mean plasma TAC/HPX levels, in this cohort. Plasma TAC/HPX levels were significantly lower in groups with Pittsburgh sleep quality index (PSQI) more than 5 (t_93_ = 2.39, *P* = 0.019), with high sleep apnoea risk (t_62.2_ = 3.32, *P* = 0.002) and with a depression score ≥ 5 (t_94_ = 2.38, *P* = 0.019). After adjusting for gender, the associations between plasma TAC/HPX levels, PSQI and high sleep apnoea risk remained statistically significant (F (1,92) = 4.49, *P* = 0.037, and F (1,95) = 6.97, P = 0.010, respectively). However, the association between TAC/HPX levels and depression score, did not remain significant after adjusting for gender (F (1,93) = 3.91, *P* = 0.051). Plasma TAC/HPX levels were significantly lower with increasing body fat percentage (F (2, 95) = 4.74, *P* = 0.011). However, this association did not remain statistically significant after adjusting for gender (F (2, 94) = 2.81, *P*≥ 0.05) (Table 1). Plasma TAC/HPX levels were significantly lower with increasing caffeine intake (F (2, 93) = 3.97, P = 0.022). This association remained statistically significant after adjusting for gender (F (2, 92) = 3.58, *P* = 0.032). A significant difference in plasma TAC/HPX levels was also observed between red meat intake tertiles (F (2, 93) = 5.55, *P* = 0.005) that remained statistically significant after adjusting for gender (F (2, 92) = 6.65, *P* = 0.002). After Bonferroni adjustment, only the association between sleep apnoea and plasma TAC/HPX levels remained statistically significant (*P* ≤ 0.002).

**Table 1:** Comparison of plasma TAC/HPX means ± SD across categories of various physiological and lifestyle components (except diet).

	Plasma TAC/HPX ± SD	*P* value
**Age (years)**		NS
< 51	0.71 ±0.25	
51–58	0.62 ± 0.21	
≥ 59	0.65 ± 0.17	
**Gender**		≤ 0.05
Male (n = 48)	0.71 ± 0.21^a^	
Female (n = 50)	0.61 ± 0.21^a^	
**Body Fat%**		≤ 0.05
< 30.83	0.75 ± 0.24	
30.83–38.70	0.61 ± 0.17	
≥ 38.71	0.61 ± 0.20	
**Sleep Quality (PSQI)**		≤ 0.05
≤ 5 (n = 68)	0.68 ± 0.22^a^	
> 5 (n = 27)	0.57 ± 0.19^a^	
**Sleep Apnoea**		≤ 0.005
Low Risk (n = 78)	0.68 ± 0.23^a^	
High Risk (n = 20)	0.56 ± 0.11^a^	
**Depression Score**		≤ 0.05
< 5 (n = 75)	0.68 ± 0.22^a^	
≥ 5 (n = 21)	0.56 ± 0.18^a^	
**Physical Activity**		NS
Low (n = 20)	0.68 ± 0.23	
Moderate (n = 43)	0.60 ± 0.18	
High (n = 35)	0.71 ± 0.23	
**Sitting Time (min/day)**		NS
< 308	0.71 ± 0.22	
308–479	0.63 ± 0.19	
≥ 480	0.63 ± 0.23	

Comparisons made using one-way ANOVA unless otherwise stated

^a^ Comparisons made using the Independent T Test

**Table 2:** Comparison of plasma TAC/HPX means ± SD across tertiles of various dietary components.

	1^st^tertile	2^nd^tertile	3^rd^tertile	*P* value
**Caffeine Intake (mg/day**)	0.74 ± 0.17	0.62 ± 0.27	0.60 ± 0.16	≤ 0.05
**Alcohol Intake (g/day)**	0.70 ± 0.23	0.60 ± 0.16	0.65 ± 0.24	NS
**Fruit Intake (g/day)**	0.59 ± 0.19	0.70 ± 0.22	0.66 ± 0.22	NS
**Vegetable Intake**	0.64 ± 0.20	0.65 ± 0.23	0.67 ± 0.21	NS
**Total Energy Intake**	0.69 ± 0.17	0.59 ± 0.22	0.69 ± 0.23	NS
**n-6 PUFA Intake (g/day)**	0.67 ± 0.23	0.67 ± 0.22	0.63 ± 0.20	NS
**n-3 PUFA Intake (g/day)**	0.65 ± 0.23	0.69 ± 0.19	0.63 ± 0.22	NS
**Saturated Fat Intake (g/day)**	0.72 ± 0.18	0.59 ± 0.21	0.66 ± 0.23	NS
**Cholesterol Intake (mg/day)**	0.70 ± 0.17	0.63 ± 0.23	0.64 ± 0.24	NS
**Red Meat Intake (g/day)**	0.75 ± 0.19	0.59 ± 0.24	0.62 ± 0.19	≤ 0.005
**Glycaemic Index**	0.64 ± 0.19	0.67 ± 0.23	0.66 ± 0.23	NS
**Retinol Intake (μg/day)**	0.69 ± 0.23	0.65 ± 0.18	0.63 ± 0.23	NS
**Vitamin E Intake (mg/day)**	0.59 ± 0.17	0.65 ± 0.20	0.70 ± 0.23	NS
**Total Carotenoid Intake (μg/day)**	0.61 ± 0.17	0.64 ± 0.20	0.70 ± 0.25	NS
**Vitamin C Intake (mg/day)**	0.62 ± 0.20	0.71 ± 0.23	0.64 ± 0.22	NS

Comparisons made using one-way ANOVA.

Caffeine Intake (mg/day) tertiles: 1^st^< 95.12, 2^nd^ 95.12–341.1, 3^rd^ ≥ 341.2; Alcohol Intake (g/day) tertiles: 1^st^: 0, 2^nd^ 0–7.6, 3^rd^ ≥ 7.7; Fruit Intake (g/day) tertiles: 1^st^< 117.87, 2^nd^ 117.87–243, 3^rd^ ≥ 244; Vegetable Intake tertiles: 1^st^<89, 2^nd^ 89–125, 3^rd^ ≥ 126; Total Energy Intake tertiles: 1^st^<5905, 2^nd^ 5905–8303, 3^rd^ ≥ 8304; n-6 PUFA Intake (g/day) tertiles: 1^st^< 7.02, 2^nd^ 7.02–11.41, 3^rd^ ≥ 11.42; n-3 PUFA Intake (g/day) tertiles: 1^st^< 1, 2^nd^ 1–1.56, 3^rd^ ≥ 1.57; Saturated Fat Intake (g/day) tertiles: 1^st^< 21.73, 2^nd^ 21.73–32.30, 3^rd^ ≥ 32.31; Cholesterol Intake (mg/day) tertiles: 1^st^<193, 2^nd^ 194–290, 3^rd^ ≥ 290; Red Meat Intake (g/day) tertiles: 1^st^< 26.40, 2^nd^ 26.40–86.80, 3^rd^ ≥ 86.80; Glycaemic Index tertiles: 1^st^ < 48.11, 2^nd^ 48.11–51.75, 3^rd^ ≥ 51.76; Retinol Intake (μg/day) tertiles: 1^st^< 208, 2^nd^ 208–357, 3^rd^ ≥ 358; Vitamin E Intake (mg/day) tertiles: 1^st^< 5.6, 2^nd^ 5.6–8.1, 3^rd^ ≥ 8.2; Total Carotenoid Intake (μg/day) tertiles: 1^st^< 8733, 2^nd^ 8733–12058, 3^rd^ ≥ 12059; Vitamin C Intake (mg/day) tertiles: 1^st^< 82, 2^nd^ 82–121, 3^rd^ ≥ 122.

There was no significant difference in mean plasma TAC/HPX between tertiles for total energy intake, or intakes of cholesterol, saturated fat, n-6 or n-3 poly unsaturated fatty acids (PUFA), vitamin E, fruits, vegetables, total carotenoid, retinol, vitamin C, ingested carbohydrates’ glycaemic index, alcohol or physical activity (P ≥ 0.05) (Table 2).

### Associations between plasma TAC/HPX levels and biochemical markers

A statistically significant non-linear association was observed between levels of plasma TAC/HPX and FPG (*r*_*s*_(98) = -0.21, *P* = 0.035). However, this association did not remain significant after adjusting for age, gender and total body fat percentage (t(93) = -0.75, *P* ≥ 0.05, *R*^*2*^ = 0.09). Plasma TAC/HPX levels were significantly associated with levels of plasma total cholesterol (*r*(98) = -0.23, *P* = 0.022) and LDL-C (*r*(98) = -0.21, *P* = 0.036). Both of these associations remained significant after adjusting for age, gender and total body fat percentage (t(93) = -2.08, *P* = 0.040, *R*^*2*^ = 0.12, and t(93) = - 2.27, *P* = 0.026, *R*^*2*^ = 0.13, respectively). No association was observed between levels of plasma TAC/HPX and HDL-C (*r*(98) = -0.11, *P* ≥ 0.05) and TG (*r*_*s*_(95) = - 0.17, *P* ≥ 0.05). A significant non-linear association was observed between plasma TAC/HPX levels and IL-1β (*r*_*s*_(96) = - 0.28, *P* = 0.005). This association remained significant after adjusting for age, gender and total body fat percentage (t(91) = -2.26, *P* = 0.026, *R*^*2*^ = 0.13). Furthermore, a statistically significant association was observed between plasma TAC/HPX levels and CRP (*r*_*s*_(96) = -0.30, *P* = 0.003) that did not remain statistically significant after adjusting for age, gender and total body fat percentage (t(91) = -1.40, *P* ≥ 0.05, *R*^*2*^ = 0.11). No association was observed between levels of plasma TAC/HPX and the inflammatory cytokines TNF- α (*r*(97) = -0.03, *P* ≥ 0.05) and IL-6 (*r*(96) = -0.16, *P* ≥ 0.05).

### Association between plasma TAC/HPX levels, blood pressure, and Framingham risk score

**Fig 1:**
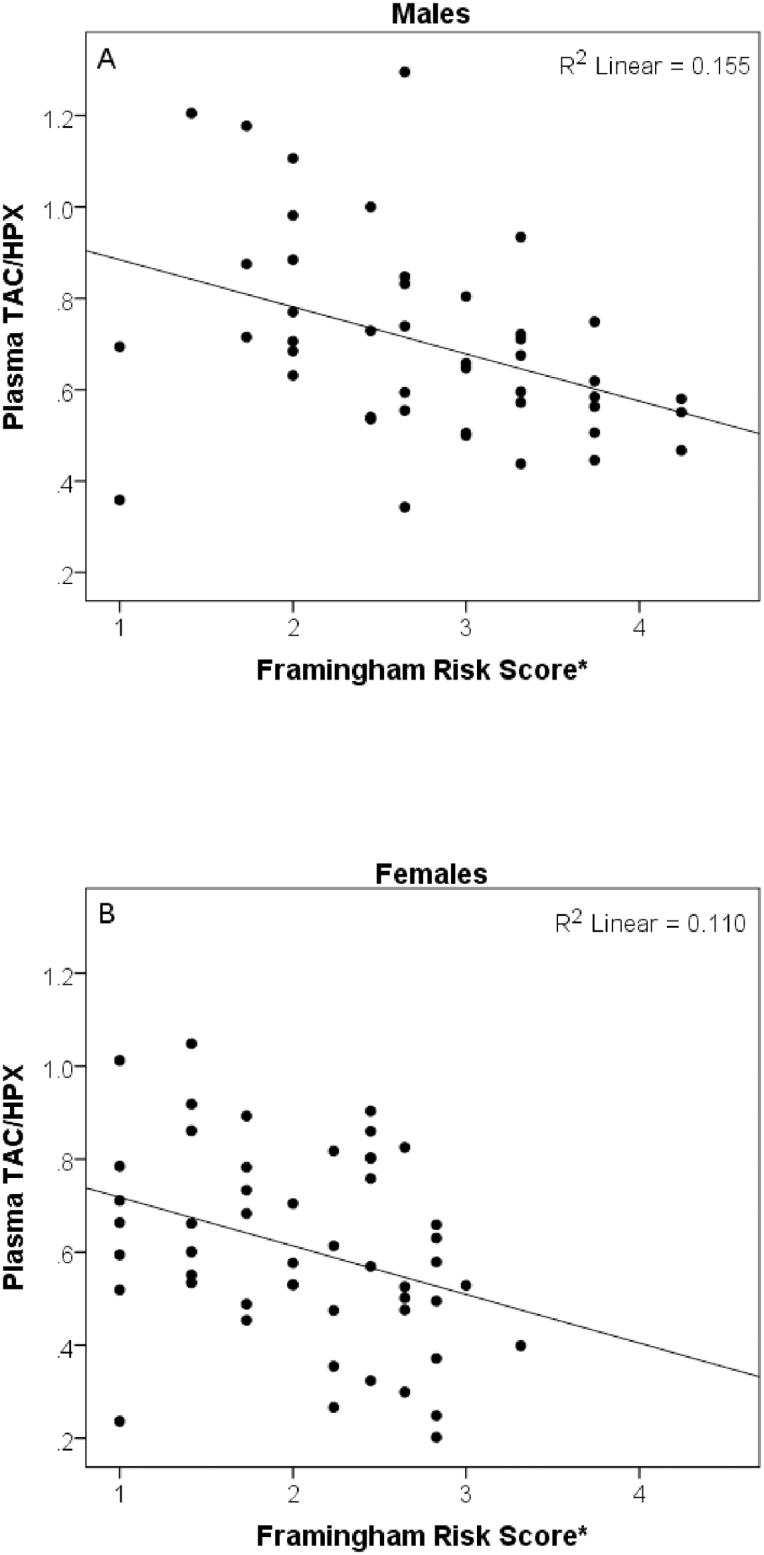
Associations between plasma total antioxidant capacity to lipid hydroperoxides ratio (TAC/HPX) levels and the 10-year risk of Framingham risk score in (A) Males (B) Females. *Data represented as square root Framingham risk score.

No significant association was observed between plasma TAC/HPX levels and systolic blood pressure (r(98) = -0.15, *P* ≥ 0.05) and diastolic blood pressure (r(98) = -0.04, *P* ≥ 0.05). However, a statistically significant association was observed between plasma TAC/HPX levels and the Framingham risk score in both males (r(45) = -0.39, *P* = 0.008) and females (r(50) = -0.33, *P* = 0.019) (Fig 1). This association remained statistically significant after adjusting for other Framingham risk score components including age, gender, LDL-C, HDL-C, systolic and diastolic blood pressures (t(87) = -2.70, *P* = 0.008, *R*^*2*^ = 0.78).

### Association between plasma TAC/HPX levels and the number of lifestyle-related risk factors

**Fig 2:**
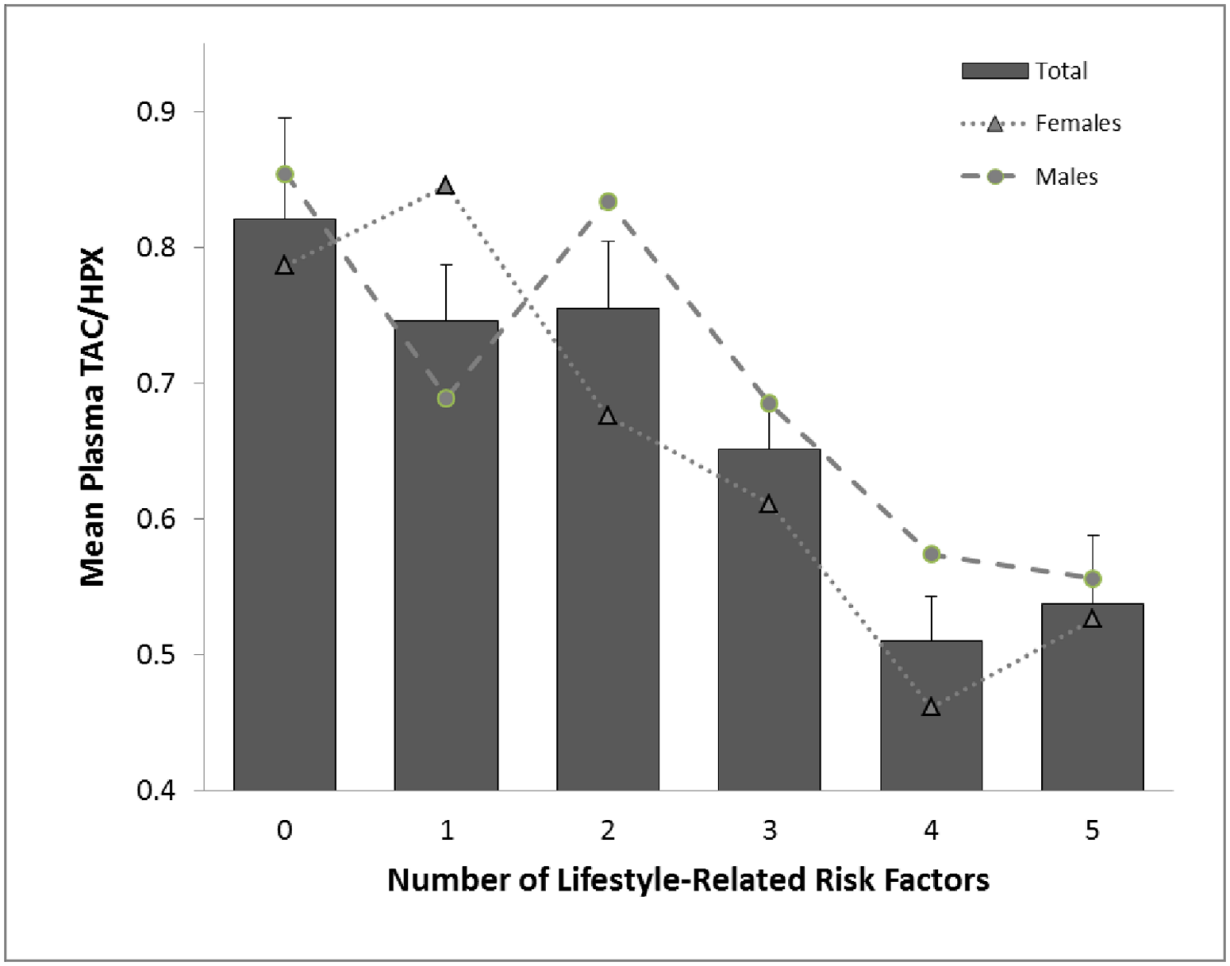
Plasma TAC/HPX mean in groups with various numbers of lifestyle-related risk factors. Data are presented as mean ± SEM. Lifestyle-related risk factors include: PSQI > 5, high sleep apnoea risk, depression score ≥ 5, red meat intake ≥ 26.40 g/day, caffeine intake ≥ 95.12 mg/day, body fat (%) ≥ 30.83. Group 0: subjects without any risk factor (n = 8), group 1: subjects with any one risk factor (n = 11), group 2: subjects with any two risk factors (n = 24), group 3: subjects with any three risk factors (n = 22), group 4: subjects with any four risk factors (n = 23), group 5: subjects with any five risk factors (n = 8). In the bar graph (both male & female), mean TAC/HPX levels were significantly lower in groups with any three risk factors compared to groups with no risk factors (*P* = 0.034); Mean TAC/HPX levels were significantly lower in groups with any four risk factors compared to groups with no risk factors (*P ≤ 0*.*001*), with any one risk factor (*P≤ 0*.*001*), with any two risk factors (*P ≤ 0*.*001*) and with any three risk factors (*P* = 0.014); Mean TAC/HPX levels were significantly lower in groups with any five risk factors compared to groups with no risk factors (*P* ≤ 0.004), with any one risk factor (*P* = 0.020) and with any two risk factors (*P* = 0.006).

There was a statistically significant and progressive decrease in plasma TAC/HPX means with increasing numbers of lifestyle-related risk factors as determined by one-way ANOVA (F (5, 90) = 6.32, *P* ≤ 0.001). Also, gender-specific analysis showed a statistically significant decrease in plasma TAC/HPX means in both males (*Welch’s* F (5, 13.04) = 3.04, *P* = 0.049) and females (F (5, 42) = 4.53, *P* = 0.002)with increasing numbers of lifestyle-related risk factors (Fig 2).

## Discussion

Oxidative stress (OS) is a recognized player in the progressive cell damage associated with development of NCDs [6, 7, 8]. The prevalence of NCDs may therefore be more effectively minimized if the biochemical abnormalities associated with OS are detected before advanced cell damage and irreversible tissue destruction occur. OS is defined as a state of redox imbalance between an organ’s exposure to reactive oxygen species (ROS) and the body’s compensatory antioxidant response [24]. As OS can arise from either an increase in ROS production and/or a decrease in antioxidant capacity, it is more suitable that monitoring of OS involves measurement of not only ROS but also antioxidant capacity at the same time.

In this study, we measured lipid hydroperoxide (HPX) levels in concert with a measure of total antioxidant capacity (TAC). Lipid HPXs are the primary products of oxidative damage to lipids which are ubiquitous in living tissue and are a very sensitive predictor of OS [25]. TAC is a measure of the cumulative antioxidant potential present in plasma, providing an index of relative systemic antioxidant capacity [26]. Considered as a related function, the TAC/HPX provides a more useful measure of redox balance than either TAC or HPX alone. Although redox balance is affected by a complex system of endogenous mechanisms, it is also significantly influenced by modifiable exogenous factors related to lifestyle [12]. As the interplay between these factors is specific to each individual, the TAC/HPX measure may provide a personalized indicator of how well an individual’s body is responding biochemically to their unique combination of lifestyle behaviours.

Much of the literature addressing the association between OS and its related complications has focused on the measurement of individual components of ROS, or antioxdiants in diseased populations [6, 7, 27]. However, to the best of our knowledge, no study has yet assessed the relationship between redox state and lifestyle behaviours in an apparently healthy population. Therefore, in this study, we investigated for the first time links between the redox measure (plasma TAC/HPX) and modifiable behaviours and, a population-based risk score (i.e. Framingham risk score) in an apparently healthy population.

In this study we observed that plasma TAC/HPX levels were significantly higher in males compared to females. Consistent with this, hydroperoxide levels have been previously reported to be higher in females compared to males. No significant difference was observed in antioxidants levels between genders [27]. The hormone estrogen may be a primary player in this observation. Despite having some antioxidant activity [28], estrogen has been shown to induce lipid peroxidation during its metabolic activation [29] and can also enhance mitochondrial ROS production [30]. In addition, since our data showed a significant negative association between body fat percentage and plasma TAC/HPX levels, the observed lower TAC/HPX in females may also be related to their significantly higher body fat percentage compared to males (data in S1 Table).

Key findings from our study also include, lower plasma TAC/HPX levels in the group with sleep disorder (higher PSQI value), and the group with higher sleep apnoea risk which were both independent of gender. Others have shown higher lipid peroxidation and lower antioxidant enzymes levels or TAC values in postmenopausal women with sleep disorders [31], and in subjects with obstructive sleep apnea [32, 33]. It has been hypothesized that good sleep promotes a state where the damage generated by ROS during waking hours can be efficiently removed [34]. Furthermore, nocturnal intermittent hypoxia has been shown to induce OS [35].

A higher depression score was also associated with lower TAC/HPX levels in our study. Though, this association did not remain significant after adjusting for gender. Similar studies have also reported a significant increase in lipid peroxidation biomarkers levels and a decrease in TAC levels with depression [36, 37].

While we observed no significant difference in mean plasma TAC/HPX between tertiles of total energy intake, we did observe that plasma TAC/HPX levels decreased with increasing body fat percentage. Although this association did not remain statistically significant after adjusting for gender, in similar studies a negative association was reported between TAC and total body fat in obese subjects [38], and a positive association between HPX and total body fat in women [39].

Interestingly, our data showed that plasma TAC/HPX levels decreased with increasing caffeine intake, which was independent of gender. Consistent with this finding, it has been reported that caffeine can increase lipid oxidation in young men [40]. In addition caffeine intake prior to exercise in men was observed to enhance exercise-induced lipid peroxidation and to decrease TAC, 24 hours and immediately after exercise, respectively [41].

A significant decrease in plasma TAC/HPX levels was observed with increasing red meat intake, which was independent of gender. In agreement with this finding, in a population-based study, Romeu et al. reported that meat intake was positively associated with lipid peroxidation biomarkers and was negatively associated with antioxidant capacity [42]. This effect may be attributed, at least in part, to the cumulative pro-oxidant properties of heme-iron and saturated fats present in red meat [42, 43].

Somewhat surprisingly, our data showed no significant differences in mean plasma TAC/HPX between tertiles for alcohol intake. Consistent with this finding, one study also reported that regular consumption of alcoholic red wine did not have any effect on plasma antioxidant capacity in healthy subjects [44], while another study indicated that moderate alcohol intake along with a fat-enriched diet did not affect the baseline concentrations of lipid peroxides [45]. Although other studies have shown an increase in OS biomarkers with alcohol treatment [46], the amount of alcohol consumed by subjects in our cohort may be too low (mean = 7.6 g/day) to affect antioxidants and OS biomarkers level significantly.

Somewhat unexpectedly, we observed no significant association between plasma TAC/HPX levels and the intake of foods and nutrients with high dietary TAC values including fruits, vegetables, vitamin E, total carotenoid, retinol and vitamin C. However, Pellegrini et al. also reported no significant association between diet and plasma values of TAC [47]. Relevant to this, it has been suggested that the TAC values for foods do not translate directly into enhanced human antioxidant defenses due to their modifications during metabolism and biokinetics [48].

There was no significant difference in mean plasma TAC/HPX between tertiles of physical activity and sitting time. Despite evidence for the effect of physical activity on promoting endogenous antioxidant defense system [49], there are reports that both acute and chronic exercise can increase plasma lipid peroxidation [50] potentially modulating the increase in TAC and any difference in TAC/HPX levels between tertiles.

While we observed no significant association between mean plasma TAC/HPX and ingested carbohydrates, a negative non-linear association was observed between levels of plasma TAC/HPX and plasma glucose. Consistent with this finding, it has been shown that lipid peroxidation increases and plasma TAC decreases in pre-diabetics compared to non-diabetics [51, 52].

While our results showed no significant difference in mean plasma TAC/HPX between intake tertiles of cholesterol, plasma TAC/HPX levels were negatively associated with plasma total cholesterol and LDL-C concentrations. As suggested by a similar finding in hyperlipidemic subjects, this is consistent with facilitated lipid peroxidation due to high availability of oxidizable lipid substrates [53].

HDL-C is the main carrier of lipid hydroperoxides in plasma and consistent with other studies [54], we observed a positive association between HDL-C and lipid hydroperoxides (data in S2 Table), though no association with TAC/HPX. Consistent with the observations of others [55], we observed no significant association between plasma levels of TAC/HPX and TG.

It is recognized that increased inflammatory activity is a primary driver of oxidative damage [56, 57]. Consistent with this widely held view [57, 58], we observed a negative non-linear association between plasma TAC/HPX levels and the inflammatory cytokine,IL-1β, independent of age, gender and total body fat percentage and a negative association between plasma TAC/HPX levels and the global inflammatory marker, CRP.

While identifying associations between individual risk factors and redox balance is valuable we were most interested in gauging whether the TAC/HPX measure of redox balance had potential as an early indicator of disease. We were therefore interested to find a strong negative association between plasma TAC/HPX levels and the Framingham Risk Score in both males and females that was independent of the Framingham risk score’s components. This result strongly supports the view that TAC/HPX should be further investigated as a potential predictor for NCDs such as cardiovascular disease. While no study has yet reported an association between TAC/HPX and Framingham risk score other investigators have reported that adding the lipid peroxidation biomarkers (e.g. 9-HETE and F2-Isoprostane) to the standard Framingham risk score significantly improved its ability to predict coronary heart disease [9].

In further support of a role for a redox balance indicator as a sensitive reflector of poor lifestyle behaviors, we observed a significant and progressive decline in mean plasma TAC/HPX with increasing numbers of lifestyle-related risk factors (Fig 2). The TAC/HPX therefore appears to serve as a sensitive biomarker for early changes in the body’s redox balance in response to unhealthy lifestyle behaviours.

While we believe that the data presented is both thorough and informative, we acknowledge that some limitations exist. First, the cross-sectional study design does not allow us to confirm causality and longitudinal studies are needed to further support these findings. Second, the relatively small number of subjects reduces the sensitivity of the study so that some relationships may have gone unnoticed and after adjusting *P* values to take into account multiple comparisons, chance findings may exist. Therefore future studies overcoming these limitations are recommended to verify the consistency of our observations.

In conclusion, the TAC/HPX ratio is significantly associated with various physiological and lifestyle components including gender, total body fat percentage, sleep quality, depression score and nutrition (meat intake and caffeine intake). Furthermore, we demonstrated that is also significantly correlated with the Framingham risk score and responds in a significant declining trend to increasing numbers of lifestyle-related risk factors. Therefore, it may serve as a robust, independent and sensitive biomarker of how an individual’s biochemistry is responding to their specific grouping of lifestyle challenges and thus whether their trajectory is toward development of a NCD.

## Supporting information

S1 Table Comparison of body fat% means ± SD across genders. (DOCX)

S2 Table Correlation coefficients for the association between plasma HDL-C and lipid hydroperoxides. (DOCX)

## Abbreviations

CRP: C—reactive protein
DXA: Dual-energy X-ray absorptiometry
FPG: Fasting plasma glucose
HDL-C: High density lipoprotein cholesterol
HPX: Hydroperoxide
IL-1β: Interleukin-1β
IL-6: Interleukin-6
LDL-C: Low density lipoprotein cholesterol
NCDs: Non-communicable disease
OS: Oxidative stress
ROS: Reactive oxygen species
TAC: Total antioxidant capacity
TC: Total cholesterol
TG: Triglyceride
TNF-α: Tumor necrosis factor- α
