# Supplementary material for: Significant relationships between a simple marker of redox balance and lifestyle behaviours; Relevance to the Framingham risk score
Source: PLoS One. 2017 Nov 6;12(11):e0187713. doi: 10.1371/journal.pone.0187713 (PMC5673171; doi:10.1371/journal.pone.0187713)
Supplement: S1 Table — (DOCX) [file pone.0187713.s001.docx]

S1 Table - Comparison of body fat% means ± SD across genders

| **Gender** | **Body Fat%** | ***P* value** |
| --- | --- | --- |
| Male (n=48) | 28.98 ± 7.30 | ≤ 0.001 |
| Female (n=50) | 39.56 ± 7.75 |  |

Comparisons made using the Independent T Test
