# Supplementary material for: Significant relationships between a simple marker of redox balance and lifestyle behaviours; Relevance to the Framingham risk score
Source: PLoS One. 2017 Nov 6;12(11):e0187713. doi: 10.1371/journal.pone.0187713 (PMC5673171; doi:10.1371/journal.pone.0187713)
Supplement: S2 Table — (DOCX) [file pone.0187713.s002.docx]

S2 Table - Correlation coefficients for the association between plasma HDL-C and lipid hydroperoxides

| **Plasma biomarker** | **Correlation Coefficient** | ***P* value** |
| --- | --- | --- |
| HDL-C (mmol/L) | 0.31 | ≤ 0.005 |
